# The national maternal near miss surveillance in China: A facility-based surveillance system covered 30 provinces

**DOI:** 10.1097/MD.0000000000017679

**Published:** 2019-11-01

**Authors:** Yi Mu, Xiaodong Wang, Xiaohong Li, Zheng Liu, Mingrong Li, Yanping Wang, Qi Li, Kui Deng, Jun Zhu, Juan Liang

**Affiliations:** a National Office for Maternal and Child Health Surveillance of China West China Second University Hospital, Sichuan University, Chengdu, Sichuan; b Department of Obstetrics, West China Second University Hospital, Sichuan University, Chengdu, Sichuan; c Key Laboratory of Birth Defects and Related Diseases of Women and Children (Sichuan University), Ministry of Education, China.

**Keywords:** China, maternal death, maternal near miss, pregnancy, surveillance

## Abstract

To introduce the National Maternal Near Miss Surveillance System (NMNMSS) in detail and to report the composition of maternal near miss (MNM) in China.

The NMNMSS was established by the National Health Commission at the end of 2010, covered over 400 health facilities from 30 provinces in China. The NMNMSS was designed to collect individual information for every pregnant woman admitted to obstetric department in the sampled health facilities. Cross tabulations and correlations were used to describe the distribution of population and sampled facilities in the NMNMSS, and to calculate the MNM mortality ratio for different complications and organ dysfunctions.

The individual survey forms of 9,051,638 pregnant women were collected in the NMNMSS between 2012 and 2017. Compared with urban areas, there are very few well-quality medical resources in rural areas. Most women with pregnancy complications in rural areas can only be treated in Level 2 and lower hospitals. MNM in women with indirect obstetric complications received treatment more frequently in Level 3 hospital. The most common maternal complications in severe maternal outcomes (including maternal near miss and maternal death) are obstetrics hemorrhage (58.7%), hypertension disorder (28.0%), and severe anemia (20.6%). The overall MNM mortality ratio is 38:1. The MNM mortality ratios are lowest in amniotic fluid embolism, HIV/AIDS, heart disease, thrombophlebitis, and sepsis. For different organ dysfunctions, the ranks of the MNM mortality ratio from low to high are renal dysfunction, respiratory dysfunction, cardiovascular dysfunction, hepatic dysfunction, neurologic dysfunction, uterine dysfunction, coagulation dysfunction.

The NMNMSS is a well-established hospital-based surveillance system for maternal complications in China. It can identify the maternal complications that need to improve health care immediately in China through a powerful longitudinal real-world evidence.

## 1 Introduction

Maternal near miss (MNM), defined as a woman who experienced a severe disease during pregnancy, childbirth or postpartum but was lucky to survive,^[1]^ can provide valuable information on obstetric care as same as maternal death.^[3,20]^ However, in the period when the concept of MNM was just proposed, there was a lack of uniform definition and identification criteria.^[4–6]^ The comparison of MNM among institutions, regions or countries is difficult due to variations in case-identification criteria.^[2]^ World Health Organization (WHO) summarized three different approaches for identifying MNM: disease-specific criteria, management-specific criteria, and organ-system dysfunction/failure based criteria,^[2]^ and proposed a unified definition and identification criteria for MNM in 2009 .^[3]^ WHO also established a guide for the implementation of monitoring MNM.^[7]^ The identification criteria for MNM include clinical criteria, laboratory markers and management based proxies related to seven dysfunctional systems (cardiovascular, respiratory, renal, coagulation, hepatic, neurologic and uterine).^[7]^ The introduction of uniform definitions and identification criteria has cleared the way for the further promotion of the concept of MNM. Since then, more and more studies on MNM have been published.^[8–10]^ In addition, the WHO has carried out a multi-country survey project focused on MNM.^[11]^ Most of these studies are cross-section studies, and mainly focus on the prevalence of the specific morbidities, the incidence and risk factors of MNM and adverse perinatal outcomes, obstetric quality and improvement strategies.

The Chinese government has taken positive measures to achieve Millennium Development Goal 5 (MDG5) in recent 20 years.^[12]^ The maternal mortality ratio (MMR) in China has declined significantly and finally achieved MDG5.^[13,14]^ However, similar to developed countries, when the MMR fell to a very low level, useful information obtained from maternal deaths was limited,^[3,20]^ and the MMR became unstable. In order to find more information to improve maternal health, and further reduce maternal mortality and improve women's quality of life, Chinese obstetrics experts have shifted their focus from maternal death to MNM.^[15]^ With financial support from the National Health Commission and technical guidance from the World Health Organization, China officially launched the National Maternal Near Miss Surveillance System (NMNMSS) at the end of 2010. In recent years, a lot of institutional, provincial and national studies have been published using the data collected from NMNMSS.^[16–19]^ However, there is a lack of systematic and comprehensive description on sampling, data collection tools and methods, and quality control for this system. The aim of this study is to introduce NMNMSS in detail and to report the composition of MNM in China.

## 2 Materials and methods

### 2.1 Sampling and settings

**Figure 1:**
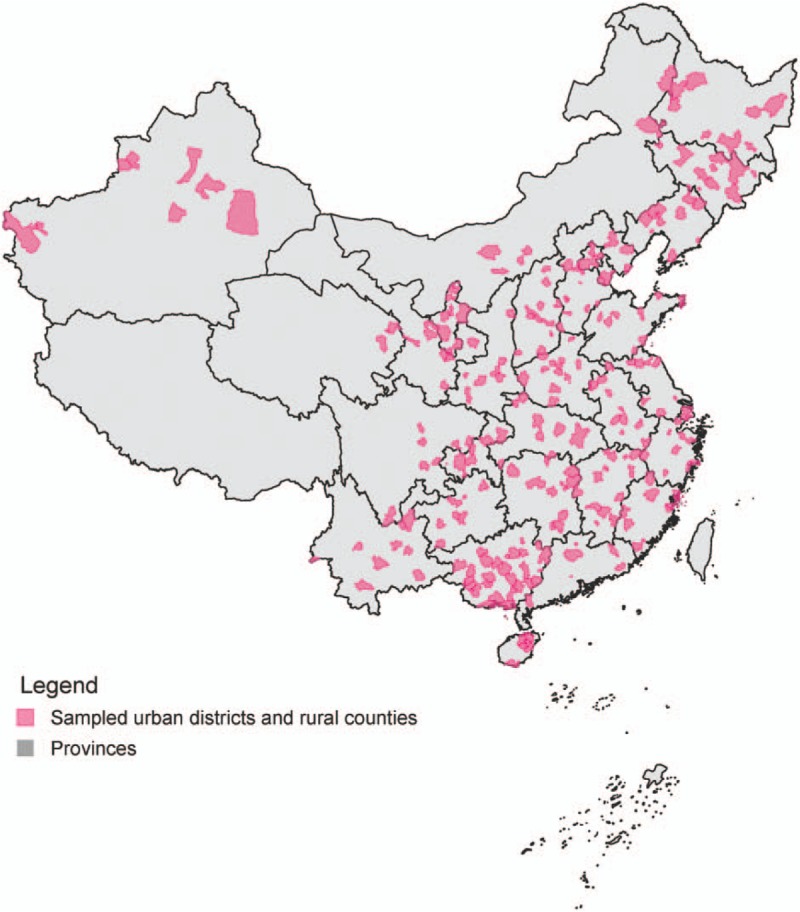
Map of sampled surveillance sites in the National Maternal Near Miss Surveillance System in mainland China.

The sampling urban districts and rural counties of the NMNMSS, which the surveillance health facilities located in, were based on China's National Maternal and Child Health Surveillance System (NMCHSS) and Provincial-level Maternal and Child Health Surveillance Systems (PMCHSSs). The NMCHSS was a well-established population-based maternal and child death and hospital-based birth defect registry system set up by the Ministry of Health of China in 1996. Thirty-seven cities (with 97 urban districts) and 79 rural counties included in the NMCHSS were stratified randomly sampled on the basis of 17 socioeconomic strata across China.^[20]^ With the reduction of maternal and child mortality rates in China, surveillance sites of the MCHSS were expanded in 2006 in order to ensure national representation. With the addition of 30 urban districts and 130 rural counties, the NMCHSS eventually covered 127 urban districts and 209 rural counties. The PMCHSSs are similar to the NMNMSS, but they are established by each provincial health administrative departments in 31 provinces in mainland China. The PMCHSSs covered all the urban districts and rural counties of the provinces, or randomly selected a part of urban districts and rural counties to represent the provinces. The surveillance sites of NMNMSS (326 urban districts and rural counties) were sampled randomly from combined NMCHSS and PMCHSSs within strata to ensure proportional representation of urban and rural populations across eastern, central, and western regions in China (Fig. 1). Refer to the health facility selection criteria of WHO Global Survey for monitoring maternal and perinatal,^[21]^ once the surveillance sites are selected, two public health facilities located in these areas with more than 1000 deliveries per year are randomly selected (or one facility if only one was available). All the hospitals in Tibet were excluded due to the lack of skilled surveillance staff. The NMNMSS covered 461 health facilities at the beginning. 20 and 3 health facilities dropped out in 2011 and 2013, respectively. Ethical approval for the NMNMSS is approved by the Ethics Committee of West China Second University Hospital, Sichuan University, China, and followed the tenets of the *Declaration of Helsinki*.

### 2.2 Data collection

In each sampled health facility, data collection began when a pregnant woman was hospitalized in obstetrics department, until she was discharged from hospital or left obstetric department. An adapted individual survey form was used to collected information on sociodemographic characteristics, pregnancy complications and terminations, interventions and process indicators, and maternal and perinatal outcomes. This individual survey form was modified according to the WHO Multicountry Survey on Maternal and Newborn Health.^[22]^ After several rounds of expert seminars, some of the elements that were difficult to collect during obstetric hospitalization in China were removed from the original form. Appendix file showed the main information collected in the individual survey form used in NMNMSS. Data were collected by obstetricians or nurses responsible for patient care mainly through the medical records review. When the individual survey form was completed, data were entered onto a web-based online reporting system centralized at the National Office for Maternal and Child Health Surveillance of China (NOMCHS). Obstetricians or nurses in sampled health facilities received trainings at the hospital level, county level, municipal level and national level before they participated in the surveillance work. As the online reporting system was upgraded in 2012 and 2016, the national trainings were implemented by NOMCHS in 2010, 2012, and 2016, respectively.

### 2.3 Definitions

Pregnancy complications and perinatal period were defined refer to Obstetrics and Gynecology textbooks (7 edition, 8 edition and 9 edition) used in China. Pregnancy complications were classified hierarchically into mutually exclusive categories of direct obstetric complications, indirect obstetric complications, and none of the above. Direct obstetric complications were abortion, ectopic, ruptured uterus, placenta praevia, placenta accreta, increta, or percreta, Abruptio placenta, soft birth canal lacerations, uterine atony, retained placenta, unspecified obstetric hemorrhage, chronic hypertension, gestational hypertension, preeclampsia, eclampsia, HELLP syndrome, puerperal infection, caesarean wound infection, premature rupture of membranes, too much or too little amniotic fluid, amniotic fluid embolism, pregnancy spit or any fetal malpresentation (breech, shoulder, or other). Indirect obstetric complications were heart disease, embolism or thrombophlebitis, hepatic disease, anemia (hemoglobin concentration of <110 g/L), renal disease (including urinary tract infection), lung disease (including upper respiratory tract infection), HIV/AIDS, connective tissue disorders, gestational diabetes mellitus, intrahepatic cholestasis of pregnancy, hypothyroidism, appendicitis, pancreatitis, Sexually transmitted diseases and cancer. The definitions and criteria of maternal death and near miss were fully consistent with the recommendation from WHO.^[7,30]^ Hospital level (level 1–3) was defined based on the size of hospital (number of beds, number of doctors and number of equipment) and capacity of medical services.^[23]^ Level 1 was the lowest. Urban districts, rural counties and regions (western, central, and eastern) were defined according to the standard definitions from National Bureau of Statistics of China.^[24]^

### 2.4 Quality control procedures

Each sampled health facility was asked to assign an associate director to supervise the progress of surveillance work. The directors randomly sampled and checked up a percentage of individual survey forms and focuses on the number of reporting pregnant women and the accuracy and completeness of forms registry. The mistakes were corrected immediately once they were found. Logical checks were also available in the web-based online reporting system. In addition, there were multiple levels of quality control in the NMNMSS. County-level quality control was implemented half a year, municipal, provincial and national quality controls were implemented once a year, respectively. County-level, municipal and provincial quality control covered all the sampled health facilities within local area, implemented by the health workers from Maternal and Child Health hospitals in each level. For the national quality control, the NOMCHS visited a random sample of six to eight hospitals in six sampled provinces once a year. All quality control results were entered onto the web-based online reporting system. Once the error exceeded the predefined standard (e.g., pregnancy complications were underreported over 5%, maternal deaths were underreported over 1%, and maternal near misses were underreported over 5%), the surveillance hospitals were asked to re-examine all of the data and correct all the mistakes.

### 2.5 Data analysis

The data was analyzed using Stata version 15.1 and R version 3.5.2. Cross tabulations and correlations were used to describe the distribution of population and sampled facilities in the NMNMSS, and to calculate the MNM mortality ratio (the ratio between the number of MNM cases and the number of maternal deaths) for different complications and organ dysfunctions. The “spmap” package of Stata was used to draw a map of the sampled surveillance sites. The “ggalluvial” package of R was used to produce the alluvial diagrams.

## 3 Results

According to China's National Census 2010, the sampled surveillance sites in the NMNMSS covered nearly 13% population of China. Table 1 shows the comparison of population distribution in 6 strata among the NMCHSS, NMNMSS and the whole country. The distribution of population in the NMCHSS and the whole country is basically the same. The proportion of the population in the eastern, central and western regions of the NMNMSS is consistent with that of the whole country, but the proportion of urban population is much higher than that of the whole country, especially in the central and western regions. Table 2 shows the number of sampled health facilities stratified by regions and hospital levels. Compared with Level 2 and 3 hospitals, the proportion of Level 1 hospitals is lower. There are very few sampled Level 1 hospitals in eastern region. Of the 441 health facilities, 26 (5.9%) hospitals have no hospital level certification.

**Table 1:**
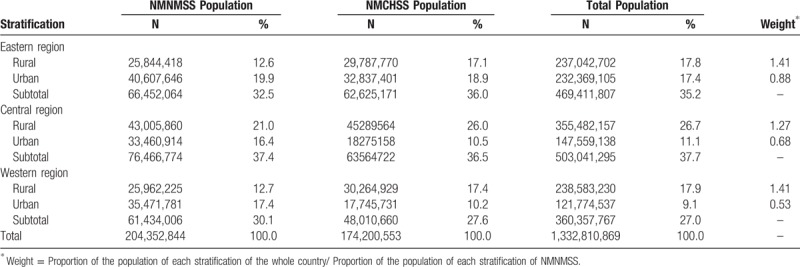
Population distribution in the National Maternal Near Miss Surveillance System (NMNMSS) and the National Maternal and Child Health Surveillance System (NMCHSS) according to China's National Census 2010.

**Table 2:**
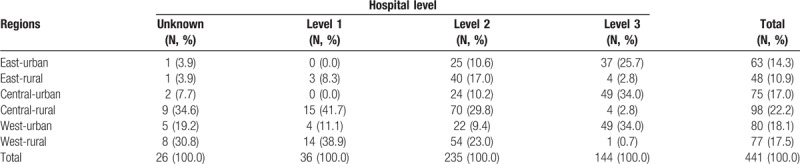
Number of sampled health facilities stratified by hospital level and regions.

**Figure 2:**
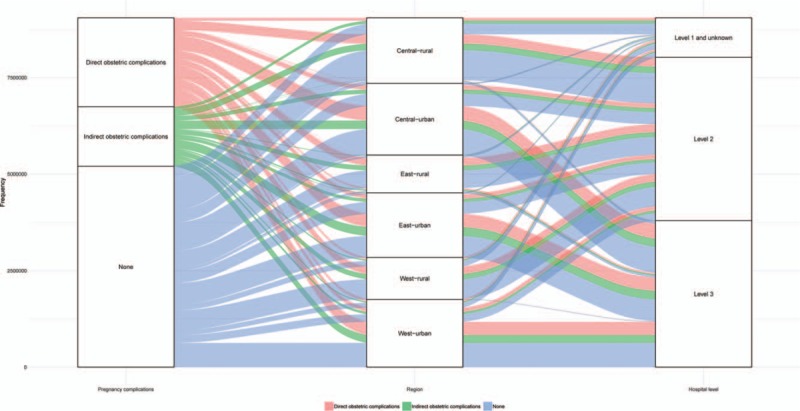
Pregnancy complications in the National Maternal Near Miss Surveillance System stratified by region and hospital level.

**Figure 3:**
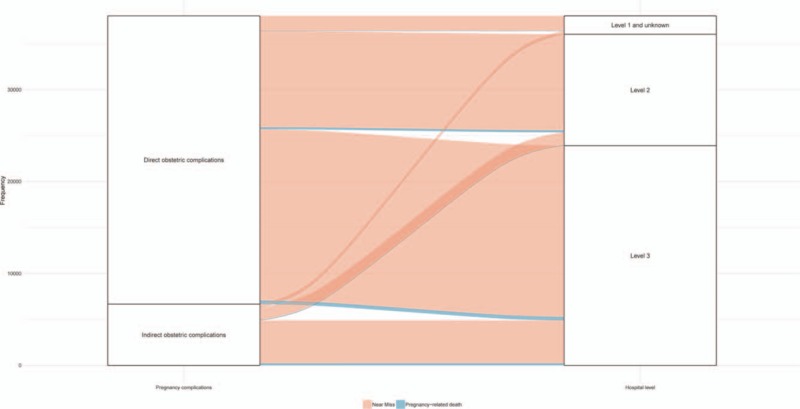
Severe maternal outcomes in the National Maternal Near Miss Surveillance System stratified by pregnancy complication and hospital level.

The individual survey forms of 9,051,638 pregnant women were collected in the NMNMSS between 2012 and 2017 in China. Level 2 and Level 3 hospitals received most of the women with pregnancy complications. Compared with urban areas, there are very few well-quality medical resources in rural areas, so most women with pregnancy complications in rural areas can only be treated in Level 2 and lower hospitals (Fig. 2). Compare with direct obstetric complications, MNM in women with indirect obstetric complications received treatment more frequently in Level 3 hospital (Fig. 3).

Table 3 shows the MNM mortality ratio in different complications and organ dysfunctions. The most common maternal complications in severe maternal outcomes (including maternal near miss and maternal death) are obstetrics hemorrhage (58.7%), hypertension disorder (28.0%), and severe anemia (20.6%). The top 3 organ dysfunctions in maternal near miss are coagulation dysfunction (65.2%), cardiovascular dysfunction (29.3%), and neurologic dysfunction (14.0%). The top 3 organ dysfunctions in maternal death are cardiovascular dysfunction (89.7%), respiratory dysfunction (73.0%), and Coagulation dysfunction (46.5%). The overall MNM mortality ratio is 38:1. For single maternal complication, the MNM mortality ratios are lowest in amniotic fluid embolism (4:1), HIV/AIDS (5:1), heart disease (7:1), thrombophlebitis (7:1) and sepsis (9:1). The MNM mortality ratios in obstetrics hemorrhage (55:1) and hypertension disorder (45:1) are higher than the average. In women with obstetrics hemorrhage, the MNM mortality ratio is lowest in soft birth canal lacerations (25:1) and highest in placenta accreta, increta, or percreta (288:1). In women with hypertension disorder, the MNM mortality ratio in HELLP syndrome is lower than in eclampsia. For different organ dysfunctions, the ranks of the MNM mortality ratio from low to high are renal dysfunction, respiratory dysfunction, cardiovascular dysfunction, hepatic dysfunction, neurologic dysfunction, uterine dysfunction, coagulation dysfunction.

**Table 3:**
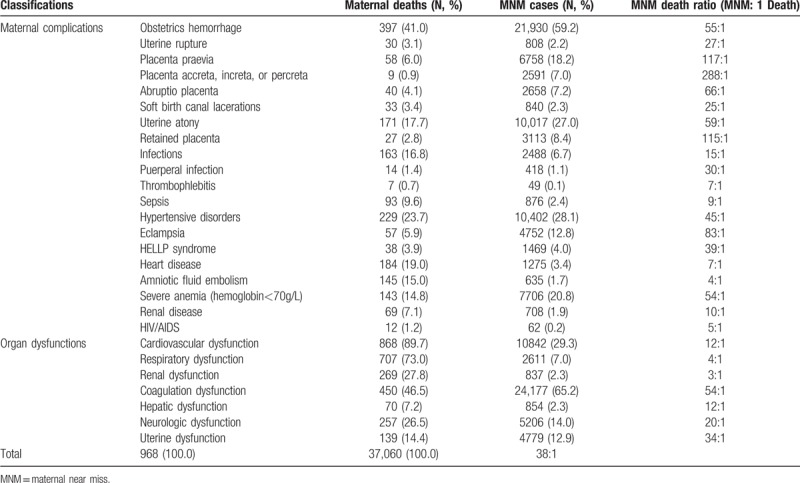
MNM mortality ratio of different complications and organ dysfunctions.

## 4 Discussion

Our study is the first time to introduce the details of the sampling and hospital distribution of China's NMNMSS. This study also identifies which maternal complications are most lacking in well-quality care in China. The NMNMSS is the largest hospital-based surveillance system for monitoring maternal complications in China. This system collects more than 1.3 million cases per year. It may also be the largest maternal complications surveillance system over the world, based on the principle of WHO Multicountry Survey on Maternal and Newborn Health.^[25]^ The previous similar studies or surveillance usually covered single hospital,^[26,27]^ or multiple hospitals with total sample size less than 50,000 per country.^[10,11,22]^ In addition, compare with these cross-sectional studies, the most important advantage of the NMNMSS is that this system can collect longitudinal data and observe the trends of maternal complications through a long-term surveillance work.

Since 2012, the hospitalized delivery rate in China was over 99%.^[28]^ Therefore, the hospital-based data is very close to population data for maternal complications. The sampled urban districts and rural counties in NMNMSS are based on the surveillance sites in NMCHSS, a nationally representative surveillance system approved by the National Health. Commission of China.^[20,28]^ However, the major problem is the health facility selection criteria in the NMNMSS. In order to ensure that a sufficient number of maternal near miss cases and maternal deaths are monitored within the hospital, large hospitals are oversampled. In particular, some hospitals in rural counties have not been sampled because of the small number of annual delivery. One of the feasible solutions to oversampling large hospitals is to weight the data against the probability of each individual to be collected in the NMNMSS in each region and rural or urban area according to the population census. Although the proportion of population in NMCHSS and whole country are very close in each stratification, the proportions of population in NMNMSS between rural and urban areas within each region are much different from those in the whole country. Therefore, we need to use the weights (see Table 1) to adjust the crude data. We have done this in some previous studies without any doubt from peer-review.^[18,19]^ A more precise approach is to calculate the weights and adjust the crude data based on the number of live births per year in each urban district or rural country. Another possible way is to adjust the crude data based on the ratio of the number of live births between the whole country and the NMNNSS stratified by maternal age group. This approach is based on the hypothesis that there is a strong association between maternal age and pregnancy complications. However, we have no way to access these two data sources in the past.

Health inequities between urban and rural areas in China have always existed.^[13]^ Although the gap in maternal mortality between urban and rural areas has gradually narrowed in recent years, our findings indicate that health resources such as high-level hospitals are still insufficient in rural areas compared with urban areas. Previous study has reported that neonatal mortality rates in urban hospitals were much lower than rural hospitals.^[20]^ The lack of well-quality medical institutions in rural areas may be one of the important reasons which is also a problem for maternal health. In recent years, the Chinese government has strengthened the management of women with severe complications and required that women with server complications must give birth in Level 3 hospitals.^[29]^ However, if the lack of level 3 hospitals in rural areas is not resolved, it will inevitably affect the accessibility of pregnant women to high-quality health care in rural areas.

According to the recommendations from WHO, higher MNM mortality ratio indicate better health care.^[7]^ The China's overall MNM mortality ratio was much higher than all the Africa countries.^[9,11,26]^ most of the Middle Eastern countries,^[22]^ and Brazil.^[8]^ And those developed countries with very low maternal mortality ratio (such as Japan and Australia) have reported a number of maternal near miss but no maternal deaths at the same time.^[11,27]^ There is still a gap in the health care and treatment for women with severe maternal complications between China and developed countries. The quality of care in indirect obstetric complications (such as heart disease) is much lower than that in most direct obstetric complications (such as obstetrics hemorrhage). Those maternal complications with lower MNM mortality ratio should be the focus in the future for improving the quality of obstetric services in China. Similarly, Chinese obstetricians should strengthen training on the rescue capabilities of renal dysfunction, respiratory dysfunction, and cardiovascular dysfunction.

There are some limitations of the NMNNSS. First, as the record of information of labor was deleted in the revised individual form used in the NMNMSS, we are unable to classify whether or not the labor was induced and whether a caesarean section is a selective caesarean section or an emergency caesarean section. This brings some limitations to the studies related to caesarean section.^[18,30]^ Second, all studies based on the data from NMNMSS are observational studies. The unobservable confounding factors cannot be controlled. So there are limitations in causal inference.

## 5 Conclusions

The NMNMSS is a well-established hospital-based surveillance system for maternal complications in China. It can identify the maternal complications that need to improve health care immediately in China. Although the large hospitals in urban areas have been oversampled in the NMNMSS, weighting the data against the probability of each individual to be collected in the system in a variety of ways may reduce the associated selection bias. Through such adjustments, the data of NMNMSS can be representative of the whole country. This will provide a powerful longitudinal real-world evidence for the study of maternal complications. In particular, the data collection in NMNMSS has covered several fertility policy stages in China (2012-2013 is the “One Child” stage, 2014 to 2015 is the “Relaxation” stage, and from 2016 to now is the “Two Children” stage). This will make it possible to assess the impact of changes in China's fertility policy on maternal and perinatal health. The data collected in NMNMSS will be a huge wealth for maternal health in China and around the world.

## Acknowledgments

We thank the institutions and staff of the National Maternal Near Miss Surveillance System for data collection.

## Author contributions

**Data curation:** Zheng Liu, Mingrong Li, Yanping Wang, Qi Li.

**Formal analysis:** Yi Mu, Xiaodong Wang.

**Methodology:** Jun Zhu, Juan Liang.

**Project administration:** Jun Zhu, Juan Liang.

**Software:** Yi Mu, Xiaohong Li, Kui Deng.

**Supervision:** Juan Liang.

**Writing – original draft:** Yi Mu.

**Writing – review & editing:** Yi Mu, Xiaodong Wang, Jun Zhu, Juan Liang.
